# Renal Function Deterioration in Postoperative (Adjuvant) Chemotherapy for Colon Cancer—Real-Life Data †

**DOI:** 10.3390/curroncol32060351

**Published:** 2025-06-13

**Authors:** Aleksandra Gładyś, Sylwia Kozak, Aleksander Jerzy Owczarek, Ewa Cedrych, Zofia Irena Niemir, Stanisław Łącki-Zynzeling, Anna Chudek, Izolda Mrochen-Domin, Iwona Gisterek-Grocholska, Jerzy Chudek

**Affiliations:** 1 Department of Internal Diseases and Oncological Chemotherapy, Medical University of Silesia in Katowice, 40-029 Katowice, Poland; aleksandragladys@outlook.com (A.G.); sylwiakozak@icloud.com (S.K.); s.lacki@outlook.com (S.Ł.-Z.); 2 Department of Oncology and Radiotherapy, Medical University of Silesia in Katowice, 40-514 Katowice, Poland; igisterek@sum.edu.pl; 3 Health Promotion and Obesity Management Unit, Department of Pathophysiology, Medical University of Silesia in Katowice, 40-752 Katowice, Poland; aowczarek@paintbox.com.pl (A.J.O.); anna.m.chudek@gmail.com (A.C.); 4 Department of Oncology, Katowice Oncology Center, 40-074 Katowice, Poland; eva.cedrych@gmail.com (E.C.); izolda.domin@wp.pl (I.M.-D.); 5 Department of Nephrology, Transplantology and Internal Diseases, University of Medical Sciences, 60-355 Poznan, Poland; zniemir@ump.edu.pl

**Keywords:** kidney function deterioration, colon cancer, nephrotoxicity, adjuvant chemotherapy

## Abstract

The knowledge concerning mild-to-moderate renal toxicity of adjuvant chemotherapy (CTH) in colon cancer patients is scarce. We retrospectively evaluated changes in the estimated glomerular filtration rate (eGFR) after three months of adjuvant treatment and the overall renal risk of the 6-month regimen in 145 patients who completed three months of therapy at three oncological centers. A decrease in eGFR of at least 1.5 mL/min/1.73 m^2^ after three months and 3.0 mL/min/1.73 m^2^ after six months was considered relevant in terms of kidney-related cardiovascular risk. Out of 114 patients who completed a 6-month regimen, kidney function deterioration occurred in 62 (54.4%) after 3 months and in 54 (47.4%) after 6 months. Age ≥ 70 years (RR = 2.66; 95% CI: 1.15–6.16) and diabetes (RR = 2.52; 95% CI: 0.98–6.45) were risk factors for kidney outcomes during the first three months of CTH. However, renal function decline during the first three months did not increase the risk of further deterioration on CTH continuation. In conclusion, older age and diabetes are factors increasing the risk of renal function deterioration during adjuvant CTH in colon cancer patients without preexisting chronic kidney disease. However, the decline during the first three months does not allow for predicting further changes under continued adjuvant therapy.

## Simple Summary

We retrospectively evaluated the estimated glomerular filtration rate (eGFR) in 145 patients with colon cancer after 3 months of adjuvant chemotherapy (aCTH) and the overall renal risk associated with this regimen continuation up to 6 months. A decrease of at least 1.5 mL/min/1.73 m^2^ in eGFR after three months and 3.0 mL/min/1.73 m^2^ after six months were considered relevant regarding kidney-related cardiovascular risk. Our findings highlight that age ≥ 70 years and diabetes were risk factors for deterioration in kidney function during the first three months of therapy. However, the decline during the first three months did not allow the prediction of further changes under continued aCTH. Thus, our research has the potential to influence clinical practice, suggesting that development of mild kidney injury should not be an argument for shortening aCTH to 3 months, even in patients with diabetes or those older than 70 without preexisting chronic kidney disease.

## 1. Introduction

Postoperative chemotherapy (CTH) for stage III and selected stage II colon cancer patients has been the established standard of care for over 30 years. Taking into account that the annual incidence rate of colon cancer exceeds 1,100,000 worldwide, and about 35% of these cases are diagnosed with stage III the burden of adjuvant CTH has a considerable impact not only on cured individuals but also on medical care providers [1]. The primary goal of post-surgery systemic treatment is to eliminate potential micrometastases, thereby reducing the risk of cancer dissemination. Total surgical resection of stage II and III colon tumors is curative in about half of patients [2]. Since predicting who will benefit from the post-surgical treatment is impossible, it should effectively reduce mortality and be as safe as possible.

The first results demonstrating the benefit of 5-fluorouracil (5-FU) in early-stage colorectal cancer after total tumor resection were published in 1990 [3]. The data from a pooled analysis of colon cancer trials (IMPACT), published in The Lancet in 1995, paved the way for introducing two 5-FU/leucovorin-based adjuvant treatment regimens as the standard of care for those patients. Compared to surgery alone, this regimen increased 3-year disease-free survival (DFS) by up to 71% and overall survival (OS) by up to 83% [4]. In the late 1990s, 5-FU and folinic acid-based regimens were established as the standard of care for adjuvant CTH worldwide. Both regimens demonstrated excellent tolerance, with a low rate of grade 4 toxicities, less than 3%. Diarrhea, stomatitis, and neutropenia were the most frequent toxicities [4]. Analysis of several clinical trials, which enrolled over 3000 colon cancer patients treated with 5-FU-based adjuvant CTH, revealed significantly better 5-year OS compared to surgery alone (71% vs. 64%, respectively), including elderly ones, with toxicity evaluation focused on nausea, vomiting, diarrhea, stomatitis, and leukopenia [5]. Other clinical studies revealed that the 22 h infusions of 5-FU were as effective as boluses but had better tolerance. Subsequently, the oral pro-drug capecitabine hit the market with comparable effectiveness and a much more convenient form of treatment [6].

Adding oxaliplatin to the infusion of 5-FU or capecitabine further improved the outcomes of OS and DFS. The updated MOSAIC trial results highlighted a favorable outcome after ten years of observation in the oxaliplatin/5-FU/folinic acid (FOLFOX4) group of patients compared to the 5-FU/leucovorin group. These findings were significantly better for the entire group and more pronounced in stage III patients (the 10-year OS rates were 67% vs. 59%, respectively; *p* = 0.016) [7].

The safety profile of oxaliplatin/capecitabine vs. 5-FU/folinic acid in adjuvant CTH for stage III colon cancer revealed less diarrhea and alopecia but more vomiting, hand–foot syndrome, and neurosensory toxicities compared to the 5-FU-treated group. Furthermore, a higher rate of grade 3/4 adverse effects (AEs), including dehydration and hypokalemia, occurred [8]. To reduce oxaliplatin toxicities, a shorter 3-month fluoropyrimidine-based regimen replaced the standard of care for low-recurrence risk of stage III colon cancer. The non-inferiority of this regimen was proved by the International Duration Evaluation of Adjuvant Chemotherapy (IDEA) pooled analysis (3-year DFS 83.1% vs. 83.9% for T1-3N1), with an additional benefit—a lower cumulative rate of neurotoxicity (16.0% vs. 44.5%) followed by improved quality of life [9].

Reducing the toxicity and sequelae of chemotherapy has become a challenge for modern oncology. Thus, the approach to balance the potential harms and benefits is a general goal for each particular patient.

The literature provides limited information concerning mild-to-moderate renal toxicity and the cardiological significance of adjuvant CTH in colon cancer patients. Thus, we evaluated renal function three months after initiating adjuvant treatment and estimated a further decline in renal function and overall renal risk associated with a 6-month adjuvant CTH.

## 2. Materials and Methods

**Figure 1:**
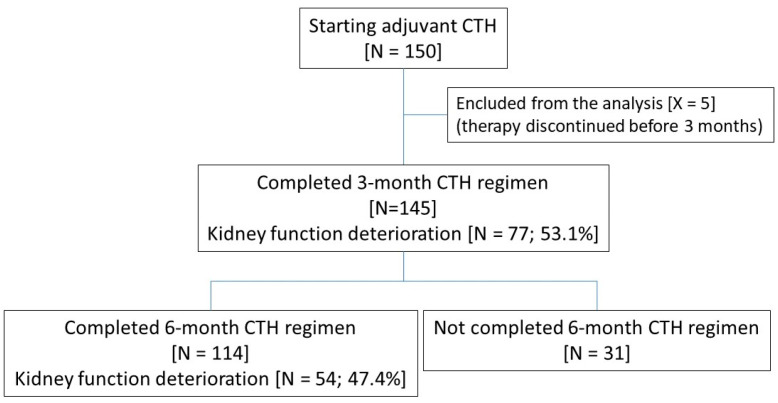
Flow chart of the analysis.

This retrospective study was conducted in accordance with the STROBE recommendations. Our analysis included 150 unselected patients who received adjuvant CTH in the postoperative management of colon cancers in three oncological centers: Department of Internal Medicine and Oncological Chemotherapy (Medical University of Silesia in Katowice), Department of Oncology and Radiotherapy (Medical University of Silesia in Katowice) and Katowice Oncology Center from 2017 to 2022. Data were retrieved from medical records. All adjuvant CTH regimens (LVFU2, FOLFOX-4, XELOX) were eligible. There were no additional criteria for enrollment. Patients who did not complete the first 3-month CTH regimen (N = 5) were excluded from the analysis (Figure 1).

The study protocol was reviewed and approved by the Bioethical Committee (PCN/CBN/0022/KB/178/21), and the requirement for informed consent was waived due to the retrospective character of the analysis.

### 2.1. Analyzed Data Set

The data set retrieved from patients’ medical records included sex, age, body mass, postoperative pathological examination, and the results of preoperative radiological imaging (computed tomography—CT of the abdomen, pelvis, and thorax (in some cases radiological examination of the chest instead of CT), clinical stage, treatment protocol of adjuvant CTH, laboratory data (including serum creatinine levels before CTH and after 3, and 6 months of CTH), and the occurrence of comorbidities (hypertension, coronary artery disease, atrial fibrillation, diabetes mellitus, chronic kidney disease).

### 2.2. Data Analysis and Study Endpoints

The estimated glomerular filtration rate (eGFR) was calculated based on the Chronic Kidney Disease Epidemiology Collaboration (CKD-EPI) formula: GFR = 141 × min (Scr/κ, 1)α × max (Scr/κ, 1) − 1.209 × 0.993 Age × 1.018 [if female], with abbreviations meaning the following: Scr, serum creatinine; κ, 0.7 for females and 0.9 for males; α, −0.329 for females and −0.411 for males; min, minimum of Scr/κ or 1; max, maximum of Scr/κ or 1 [10].

Chronic kidney disease (CKD) was scored based on the new age-adjusted thresholds of eGFR: <75 mL/min/1.73 m^2^ for younger than 40 years, 45–60 mL/min/1.73 m^2^ for those aged 40 to 65 years, and <45 mL/min/1.73 m^2^ for older than 65 [11]. An eGFR decline of cardiological significance was defined as a decrease of at least 30% from the baseline value. To be considered significant, a reduction in eGFR had to reach at least 1.5 mL/min/1.73 m^2^ for three months or at least 3.0 mL/min/1.73 m^2^ after six months. The definitions (eGFR decline > 6 mL/min/1.73 m^2^ per year) were based on surrogate endpoints for CKD progression, as suggested by Ferguson et al. [12], for analyses of eGFR decline as a continuous variable.

The lack of data regarding expected changes in eGFR precluded the calculation of the study group size.

### 2.3. Statistical Analysis

Data were entered manually into the database and cross-checked before statistical analysis was performed. There were no data imputations. To avoid attrition bias, urine analysis data were excluded from the analysis. Statistical analyses were performed using STATISTICA 13.0 PL (TIBCO Software Inc., Palo Alto, CA, USA). A *p*-value of less than 0.05 determined statistical significance. All tests were two-tailed. Imputations were not performed for missing data. Nominal and ordinal data were expressed as percentages. Interval data were expressed as the mean value ± standard deviation. The distribution of variables was evaluated using the Anderson–Darling test and a quantile–quantile (Q–Q) plot. The Levene test was used to assess the homogeneity of variances. Nominal and ordinal data were compared with the χ^2^ test. Comparisons between two groups for interval data were carried out with the Student *t*-test, while for repeated measurements, with ANOVA and contrasts as post-hoc tests. Sensitivity analyses included potential clinical risk factors for deterioration in renal function. They were presented as relative risk with 95% confidence intervals and corresponding *p*-values.

## 3. Results

### 3.1. Study Group Characteristics

Finally, the analysis included 145 of 150 patients starting adjuvant CTH for colon cancer. There was a wide age range, from 36 to 83 years (mean age 62 ± 11 years). Five patients terminated CTH before the end of the three months of therapy due to unacceptable toxicity and were excluded from further analysis (Figure 1).

Almost two-thirds of 145 patients initiated adjuvant CTH with oxaliplatin (Table 1). The 5-FU-based regimen was more frequently administered in older patients (aged ≥ 70 years) than in younger ones (62.5% vs. 29.2%; *p* < 0.001).

Of 145 subjects, 114 (78.6%) completed six-month adjuvant CTH, while 31 discontinued the therapy before the end of 6 months of treatment (Figure 1 and Table 1). The group that completed the six-month CTH comprised 21.9% of patients aged 70 years or more, 63.2% with hypertension, 17.5% with diabetes, and, unexpectedly, only two persons with CKD (i.e., eGFR below 60 mL/min/1.73 m^2^).

### 3.2. Preterm Termination of Adjuvant CTH

As mentioned above, thirty-one patients did not complete the scheduled CTH regimen due to toxicity, as reported by clinicians, primarily due to myelotoxicity, hand–foot syndrome, diarrhea (N = 28), or disease recurrence (N = 3). We want to stress that no kidney toxicity was the reason for therapy discontinuation. The characteristics of patients’ subgroups who completed and those who did not complete the 6-month scheduled CTH regimens were almost identical (Table 1). Notably, among those who discontinued the 6-month cycle, the percentage of subjects with hypertension was lower (45.2% vs. 63.2%), nearly approaching the statistical significance (*p* = 0.07).

### 3.3. Deterioration in Kidney Function During Three and 6-Month Adjuvant CTH

The mean decline in eGFR after three months in the entire group was 2.3 ± 12.4 mL/min/1.73 m^2^, corresponding to a 2.2 ± 13.5% decline compared to the initial values. A decline in eGFR > 30 percent was noted in five patients (3.4%). Four of them continued CTH. Approximately half of the patients (N = 77; 52.1%) experienced a significant decline in eGFR, defined as a decrease of greater than 1.5 mL/min/1.73 m^2^. The continuation of CTH during the subsequent three months resulted in a significantly smaller deterioration of eGFR (−3.1 ± 12.5 mL/min/1.73 m^2^ during the 6 months vs. −2.5 ± 12.7 mL/min/1.73 m^2^ after the initial 3-month regimen). We observed an eGFR decline exceeding 30% in three patients (2.6%) at the end of adjuvant chemotherapy. In nearly half (N = 47; 47.4%) of those who completed the regimen, the eGFR declined by more than 3.0 mL/min/1.73 m^2^ (Table 2).

The decline in eGFR was higher in patients aged 70 years or older after three and six months of adjuvant CTH compared to the younger group. The mean eGFR decline after three months was 5.4 ± 10.1 vs. 1.5 ± 12.8 mL/min/1.73 m^2^ (*p* = 0.12), corresponding to −6.2 ± 12.5% vs. −1.1 ± 13.6% changes of the initial kidney filtration (*p* = 0.06), in older and younger, respectively. After six months, the difference in eGFR decline was more significant—6.2 ± 8.5 vs. 2.2 ± 12.3 mL/min/1.73 m^2^ (*p* = 0.06)—and reached statistical significance when expressed in percentages of the initial eGFR: −7.7 ± 10.4% vs. −2.1 ± 12.9% (*p* < 0.05).

Overall, at the end of adjuvant CTH, only one patient (3.5%) met the CKD criteria based on the eGFR criterion.

### 3.4. A Subanalysis of Patients Who Experience Kidney Function Deterioration After 3-Month CTH

**Figure 2:**
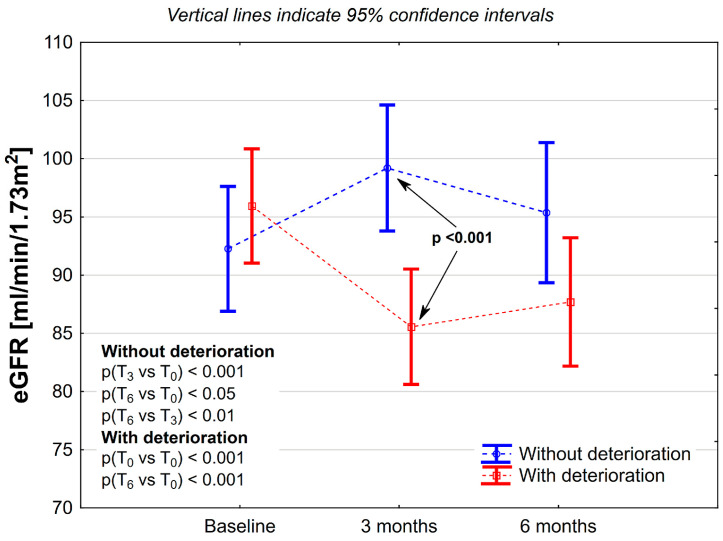
Changes in estimated glomerular filtration rates during adjuvant chemotherapy in colon cancer patients, stratified according to significant kidney function deterioration after three months (eGFR decline ≥ 1.5 mL/min/1.73 m^2^).

The mean deterioration of kidney function—eGFR decline in the subgroup was 10.4 ± 9.6 mL/min/1.73 m^2^, corresponding to 10.9 ± 9.4%. In four patients, an eGFR decline was greater than 30%. The decline after six months did not increase (8.2 ± 10.4 mL/min/1.73 m^2^), corresponding to 8.9 ± 10.4% of the initial eGFR (Figure 2). In this subgroup, 72.6% of patients met the criteria for a significant decline in eGFR, defined as a decrease of greater than 3.0 mL/min/1.73 m^2^. Additionally, a reduction in eGFR of greater than 30 percent was observed in three patients. In ANOVA with repeated measurements, there was no significant influence of the group (with/without deterioration; *p* = 0.11) on eGFR values; however, substantial changes over time (*p* < 0.05) and interaction between time and group were noted (*p* < 0.001). No changes were observed between groups at baseline (p = 0.32). Still, at the 3rd and 6th months, eGFR values were lower in the group with deterioration (*p* < 0.05 and *p* = 0.07, respectively) compared to the group without deterioration. At the 3rd and 6th months, eGFR values were significantly lower (in the group with deterioration) or higher (in the group without deterioration) than at baseline. Moreover, there was a significant difference in eGFR values between the 6th and 3rd months in the group without deterioration (*p* < 0.01). In the group with deterioration, only a tendency toward statistical significance was noted (p = 0.09)—Figure 2.

### 3.5. A Subanalysis of Patients Who Did Not Experience Kidney Function Deterioration After 3-Month CTH

In this subgroup, we observed an increase in eGFR during the first three months of CTH (6.9 ± 8.7 mL/min/1.73 m^2^), corresponding to 7.9 ± 9.6% of the initial eGFR. During the second part of the adjuvant CTH regimen, 51.9% of patients (n = 51) experienced an eGFR decline of more than 1.5 mL/min/1.73 m^2^. However, only one patient experienced an eGFR decline exceeding 3.0 mL/min/1.73 m^2^.

### 3.6. Factors Explaining Significant eGFR Decline During the 6-Month Regimen

The analysis showed that older age (≥70 years)—RR = 2.66 (95% CI: 1.15–6.16; *p* < 0.05), and the occurrence of diabetes—RR = 2.52 (95% CI: 0.98–6.45; *p* = 0.05) increased the risk of kidney function deterioration during adjuvant CTH in those who completed the regimen.

Notably, the significant deterioration of renal function during the first three months of adjuvant CTH did not increase the risk of further decline with continued therapy. Namely, the frequency of renal function impairment during the subsequent three months of adjuvant CTH was even lower in patients who had already experienced functional deterioration (during the first three months) of adjuvant CTH than in the opposite group (without deterioration during the first three months) Table 3.

## 4. Discussion and Conclusions

Postoperative oxaliplatin and fluoropyrimidine-based CTH enhances the rates of 5-year survival by up to 20% for stage III colon cancer compared to surgery alone, which yields 5-year survival rates of 45–65% and 68–83% for stages III and II, respectively [13]. Nevertheless, better survival outcomes do not accompany an improved general health condition and quality of life due to CTH-related toxicity. The deterioration of kidney function is considered an adverse event in cancer survivors. In our cohort, clinically relevant deterioration (eGFR decline > 30%) has occurred in 3.4% of patients after 3 months and in 2.6% after completing a 6-month regimen, more frequently in patients with diabetes and those 70 years old or older. Notably, four of five patients continued the adjuvant CTH, experiencing a clinically relevant decline in eGFR after 3 months. More frequently, there was a mild deterioration of eGFR of uncertain clinical relevance, diagnosed based on the change of eGFR, which was detected in approximately half of the patients receiving adjuvant CTH. Such a mild deterioration of kidney function does not generate immediate clinical consequences but may affect the frequency of cardiological outcomes in cancer survivors. The study of Zhang et al. showed that adjuvant CTH therapy increased cardiovascular mortality in patients with stage I and worsened OS in comparison to those who did not receive it (hence, international guidelines do not recommend it) [14]. However, adjuvant CTH improves 3-year OS, and even in patients aged over 70 years, the risk of cancer-related death three times outweighed the cardiovascular causes, 74.4% vs. 25.6% in stage III patients [15]. Therefore, increased kidney toxicity in patients without significant comorbidity and having good performance status cannot be considered as the cause for disqualification from adjuvant CTH.

We identified the age of over 70 years and the occurrence of diabetes as factors contributing to renal function decline during adjuvant CTH. However, the risk of worsening kidney function during the continuation of adjuvant CTH beyond the third month was difficult to predict. In addition, our study shows that oxaliplatin-based adjuvant CTH did not increase the risk of kidney function deterioration in patients without CKD before treatment.

CKD is considered one of the well-established contraindications for adjuvant CTH, including a poor performance status (according to the Eastern Cooperative Oncology Group Performance Status score—ECOG PS > 2), severe or uncontrolled infections, and comorbidities such as liver, heart, and other diseases that may potentially affect overall lifespan. Despite long-term worldwide experience with adjuvant treatment of colon cancer, the frequency of CTH-induced renal injury remains poorly defined. Clinical guidelines recommend reducing standard drug doses of chemotherapeutics to prevent the increased toxicity observed in clinical trials related to the diminished renal elimination of medical products and their metabolites. Colon cancer patients with creatinine clearance (CrCl) < 50–60 mL/min are exposed to more CTH-related AEs. Results of metastatic colorectal clinical trials showed a more favorable safety profile for capecitabine compared to 5-FU/LV in patients with moderate renal impairment (CrCl 30–50 mL/min). Notably, a higher incidence of treatment withdrawal and a higher dose reduction rate in the 5-FU/LV group compared to the capecitabine-treated group (30% vs. 25% and 52% vs. 44%, respectively) was reported, due to the occurrence of AEs influencing the disease outcome. Efficacy data in the group of patients with CKD have shown that those who required a 49–64% initial dose reduction of 5-FU/LV were at a not statistically significant but higher risk of disease progression and death (HR = 1.30; *p* = 0.19) [14]. In contrast, the efficacy of a 50–75% reduction in the initial dose of capecitabine did not alter the outcomes [16,17]. The safety profile of oxaliplatin was investigated in individuals with various stages of renal dysfunction, with no distinctive toxicity observed for CrCl beyond 20 mL/min [18].

According to National Kidney Foundation (NKF) guidelines, the estimation of GFR impairment should be based on the CKD-EPI formula and distinguish five stages of CKD [19]. The CKD-EPI formula was developed by the CKD Epidemiology Collaboration (CKD-EPI) research group, led by Andrew S. Levey, with interests in estimating GFR and evaluating surrogate endpoints for clinical trials in CKD. According to this formula, the prevalence of chronic kidney disease (CKD) in patients with colorectal cancer undergoing surgery has been reported to be approximately 10%, which corresponds to its rate in the general population (13.4% for all five CKD stages and 10.6% for stages 3–5) [20,21]. According to the literature, stages 3 to 4 CKD were the most frequent in Japan, affecting 20.1% of 1127 consecutive patients who underwent curative colorectal resection [22]. The higher prevalence of CKD in patients in this, one of the longest-living populations, may be explained by its more common occurrence among older people and those suffering from obesity and diabetes [21]. Notwithstanding, there were only 1.4% of patients with CKD in our real-world cohort, indicating the exclusion of the affected patients from adjuvant CTH, due to expected increased toxicity. However, the deterioration of eGFR after 3 months of adjuvant CTH did not confirm the criteria for a G3-4 creatinine increase (an increase over three times the baseline value) according to the Common Terminology Criteria for the AEs occurrence (CTCAE version 5.0) [23]. Consequently, four of five patients continued CTH beyond the third month, and three completed the 6-month schedule.

A potentially accepted option in patients with mild CKD or at risk of CKD development would be shortening adjuvant CTH to 3 months, based on the non-inferiority of the 3-month regimen, as proven by the IDEA pooled analysis [9], if further kidney function decline could be observed. However, our study failed to demonstrate that a mild decline in kidney function during the first 3 months of adjunctive CTH, even in patients with diabetes or those older than 70 without preexisting CKD, can predict further deterioration of eGFR upon continuation of therapy. In addition, we indirectly demonstrate a lack of a cumulative rate of nephrotoxicity when therapy is continued beyond the third month.

An essential limitation of our study is its retrospective nature, with limited control of confounding variables, and the low number of CKD patients who are especially predisposed to kidney injury during adjuvant CTH. In this context, the lack of data concerning urine analysis (not routinely performed in patients starting adjuvant CTH) requires attention. Consequently, we could not calculate the risk of albuminuria related to the therapy (which would enable a closer estimation of CKD occurrence). Moreover, our analysis did not include data on kidney function follow-up to address renal outcomes after the completion of adjuvant CTH. We acknowledge the lack of a priori sample size calculation due to the unavailability of comparable data, which may limit the statistical power of the study’s findings. Therefore, a study with a larger sample size is required to confirm our findings and provide more definitive conclusions.

In conclusion, our study identified age >70 and diabetes as factors increasing the risk of mild-to-moderate deterioration of renal function during adjuvant CTH in colon cancer patients without preexisting chronic kidney disease. The use of oxaliplatin does not increase the risk of deterioration in kidney function. Deterioration of renal function during the first three months of adjuvant CTH therapy does not allow for prediction of the risk of further reduction under continued therapy.

## Figures and Tables

**Table 1:** Characteristics of colon cancer patients starting adjuvant chemotherapy who finished a 3-month regimen, with stratification to those who completed and did not complete a 6-month therapy.

	All [N = 145]	Completed 6-Month Regimen [N = 114]	Non-Completed 6-Month Regimen [N = 31]	*p*
Women [N (%)]	76 (52.4)	61 (53.5)	15 (48.4)	0.61
Age [years]	62 ± 11	62 ± 11	63 ± 10	0.54
Age ≤50 years [N (%)]	21 (14.5)	18 (15.8)	3 (9.7)	0.57
Age ≥ 70 years [N (%)]	32 (22.1)	25 (21.9)	7 (22.6)	0.94
BMI [kg/m^2^]	26.9 ± 4.3	27.0 ± 4.3	26.5 ± 4.6	0.52
Obesity [N (%)]	33 (23.1)	26 (23.2)	7 (22.6)	0.94
Clinical stage [N (%)]				
II	32 (22.1)	25 (21.9)	7 (22.6)	0.99
III	99 (68.3)	78 (68.4)	21 (67.7)	
IV	14 (9.6)	11 (9.7)	2 (9.7)	
Chemotherapy regimen [N (%)]				
5-FU (de Gramont)	53 (36.6)	42 (36.8)	11 (35.5)	0.31
FOLFOX-4	55 (37.9)	46 (40.4)	9 (29.0)	
XELOX	37 (25.5)	26 (22.8)	11 (35.5)	
Comorbidity [N (%)]				
Hypertension	86 (59.3)	72 (63.2)	14 (45.2)	0.07
Coronary artery disease	18 (12.4)	13 (11.4)	5 (16.1)	0.48
Atrial fibrillation	6 (4.1)	5 (4.4)	1 (3.2)	0.77
Diabetes mellitus	28 (19.3)	20 (17.5)	8 (25.8)	0.30
Chronic kidney disease	2 (1.4)	2 (1.8)	0	1.00

Mean ± standard deviation or number (percentage).

**Table 2:** Kidney function before initiation and during adjuvant chemotherapy (aCTH) in colon cancer patients, with stratification to those who completed and did not complete a 6-month regimen.

	All [N = 145]	Completed 6-Month Regimen [N = 114]	Non-Completed 6-Month Regimen [N = 31]	*p*
eGFR—estimated glomerular filtration rate				
Initial [mL/min/1.73 m^2^]	93.5 ± 18.8	92.3 ± 19.5	90.6 ± 15.8	0.34
After 3-month aCTH [mL/min/1.73 m^2^]	91.1 ± 20.4	91.8 ± 20.8	88.8 ± 19.1	0.47
After 6-month aCTH [mL/min/1.73 m^2^]	–	91.2 ± 22.1	–	–
eGFR changes				
During 3-month aCTH [mL/min/1.73 m^2^]	−2.3 ± 12.4	−2.5 ± 12.7	−1.9 ± 11.5	0.81
After the third month of aCTH [%]	−2.2 ± 13.5	−2.3 ± 13.3	−2.0 ± 14.4	0.92
During 6-month aCTH [mL/min/1.73 m^2^]	–	−3.1 ± 11.6	–	–
During 6-month aCTH [%]	–	−3.3 ± 12.5	–	–
eGFR decline				
≥1.5 mL/min/1.73 m^2^ after 3-month aCTH [N (%)]	77 (52.1)	62 (54.4)	15 (48.4)	0.55
≥3.0 mL/min/1.73 m^2^ after 6-month aCTH [N (%)]	–	54 (47.4)	–	–
≥1.5 mL/min/1.73 m^2^ after the third month of aCTH [N (%)]	–	47 (41.2)	–	–
>30% after 3-month aCTH [N (%)]	5 (3.4)	4 (3.5)	1 (3.2)	0.94
>30% after 6-month aCTH [N (%)]	–	3 (2.6)	–	–

Mean ± standard deviation or number (percentage).

**Table 3:** Kidney function before and during adjuvant CTH (aCTH) in patients who completed a six-month scheduled aCTH with stratification according to significant kidney function deterioration after three months (eGFR decline ≥ 1.5 mL/min/1.73 m^2^).

	Deterioration [N = 62]	No Deterioration [N = 52]	*p*
Female [N (%)]	33 (53.2)	28 (53.8)	0.95
Age [years]			
Age ≥ 70 [N (%)]	19 (30.6)	6 (11.5)	<0.05
Obesity [N (%)]	15 (24.6)	11 (21.6)	0.71
Diabetes mellitus [N (%)]	15 (24.2)	5 (9.6)	<0.05
Chemotherapy [N (%)]			
5-FU (de Gramont)	27 (43.6)	15 (28.8)	<0.01
FOLFOX-4	7 (11.3)	19 (36.5)	
XELOX	28 (45.2)	18 (34.6)	
Estimated glomerular filtration rate—eGFR			
Initial [mL/min/1.73 m^2^]	95.9 ± 21.1	92.3 ± 17.4	0.32
After 3-month aCTH [mL/min/1.73 m^2^]	86.6 ± 20.6	99.2 ± 18.5	<0.001
After 6-month aCTH [mL/min/1.73 m^2^]	87.7 ± 23.2	95.3 ± 20.3	0.07
eGFR change			
During 3-month aCTH [mL/min/1.73 m^2^]	−10.4 ± 9.6	6.9 ± 8.7	<0.001
During 3-month aCTH [%]	−10.9 ± 9.4	7.9 ± 9.6	<0.001
During 6-month aCTH [mL/min/1.73 m^2^]	−8.2 ± 10.4	3.1 ± 10.0	<0.001
During 6-month aCTH [%]	−8.9 ± 10.4	3.4 ± 11.6	<0.001
eGFR decline			
>30% after 3-month aCTH [N (%)]	4 (6.4)	–	–
≥1.5 mL/min/1.73 m^2^ after the third month aCTH [N (%)]	20 (32.3)	27 (51.9)	<0.05
≥3.0 mL/min/1.73 m^2^ after 6-month aCTH [N (%)]	45 (72.6)	9 (17.3)	<0.001
>30% after 6-month aCTH [N (%)]	3 (4.8)	–	–
Kidney dysfunction			
After 3-month aCTH [N (%)]	4 (6.4)	1 (1.9)	0.37
After 6-month aCTH [N (%)]	1 (1.6)	0	1.00

Mean ± standard deviation or number (percentage).

## Data Availability

The data presented in this publication are available on request from the corresponding author.

## Abbreviations

aCTH	Adjuvant chemotherapy
CTH	Chemotherapy
eGFR	Estimated glomerular filtration rates
5-FU	5-fluorouracil
DFS	Disease-free survival
OS	Overall survival
FOLFOX4	Oxaliplatin/5-FU/folinic acid
CT	Computed tomography
CKD-EPI	Chronic Kidney Disease Epidemiology Collaboration
Scr	Serum creatinine
CKD	Chronic kidney disease
ECOG PS	Eastern Cooperative Oncology Group Performance Status
CrCl	Creatinine Clearance
NKF	National Kidney Foundation
CTCAE	Common Terminology Criteria for Adverse Effects
